# Sub-wavelength Laser Nanopatterning using Droplet Lenses

**DOI:** 10.1038/srep16199

**Published:** 2015-11-06

**Authors:** Martí Duocastella, Camilo Florian, Pere Serra, Alberto Diaspro

**Affiliations:** 1 Nanoscopy, Istituto Italiano di Tecnologia, Via Morego 30, 16163 Genova, Italy; 2 Departament de Física Aplicada i Òptica, Universitat de Barcelona, C/Martí i Franqués 1, 08028 Barcelona, Spain

## Abstract

When a drop of liquid falls onto a screen, e.g. a cell phone, the pixels lying underneath appear magnified. This lensing effect is a combination of the curvature and refractive index of the liquid droplet. Here, the spontaneous formation of such lenses is exploited to overcome the diffraction limit of a conventional laser direct-writing system. In particular, micro-droplets are first laser-printed at user-defined locations on a surface and they are later used as lenses to focus the same laser beam. Under conditions described herein, nanopatterns can be obtained with a reduction in spot size primarily limited by the refractive index of the liquid. This all-optics approach is demonstrated by writing arbitrary patterns with a feature size around 280 nm, about one fourth of the processing wavelength.

Optical methods are unrivaled as patterning tools thanks to the possibility to operate in ambient conditions, integration in direct-writing systems and ease of use. However, the far-field diffraction barrier limits the minimum feature size in optical systems to about half the processing wavelength, imposing a serious restriction for nanopatterning at visible or infrared wavelengths. The primary strategy to overcome diffraction has been the use of near-field effects. By focusing light through the tip of an atomic force microscope^1^, a microsphere^2^,^3^,^4^, or a plasmonic lens^5^, evanescent waves can be coupled to the material enabling deep sub-wavelength patterning. In this way, feature sizes below 100 nm can be produced, but maintaining the spacing between probe and substrate is extremely critical and difficult to perform in practice. Self-positioning systems^6^ offer an interesting solution, although they tend to be time consuming and/or require complex setups^7^. In contrast, sub-wavelength feature sizes can also be fabricated by exploiting non-linarites in the interaction of light with a particular material, as in multiphoton absorption^8^,^9^,^10^ or photopolymerization inspired by reversible saturable optical fluorescence transition (RESOLFT) microscopy^11^,^12^. Since these methods are based on the particular photophysics of the material to be patterned^13^, resolution enhancement is material-dependent and optimal results are typically constraint to a narrow range of available photoresists.

An interesting alternative for sub-wavelength optical patterning consists of using a dielectric hemispherical lens, known as a solid immersion lens (SIL), placed in contact with the surface. A SIL effectively increases the numerical aperture of a focusing optical system, producing an enhancement in lateral resolution only limited by the refractive index of the SIL material. Successful implementations of SILs include microscopy^14^,^15^,^16^, spectroscopy^17^, photolithography^18^ and optical recording^19^, with reported feature sizes as small as 100 nm^20^. However, the use of SILs for surface nanopatterning presents a series of problems that prevent this approach to be widely implemented. For instance, the relative high laser energies usually required for processing a surface, especially in the case where laser ablation is to be performed, can irreversibly damage SILs. This is particularly problematic due to the challenges involved in the fabrication of SILs. In fact, conventional grinding techniques are time consuming and are limited to SILs with diameters larger than 1 mm^21^. Small lenses are desirable since they suffer from less aberrations and they can be easily positioned at local uniform parts of an overall uneven surface. Promising methods such as photopolymerization^22^ or chemical etching^21^ can produce high-quality micro-SILs, but the fabrication process remains cumbersome. In addition, to generate nanopatterns over an extended surface one needs to mechanically translate the SIL along different positions of the sample. Such stepwise approach is inherently slow and requires sophisticated feedback control systems.

Droplet-assisted laser processing (DALP) addresses the challenges encountered in current micro and nanopatterning techniques. Our approach is based on a two-step process (Fig. 1). First, we use laser-induced forward transfer (LIFT)^23^ to print droplets with sizes ranging from 10 to 200 μm into specific areas of interest of a surface. Next, we use the printed droplets as lenses to focus laser pulses directly below each droplet, locally modifying the surface. Notably, the same laser source used for printing can be used for surface patterning. One can consider the droplet to act as the liquid version of SILs without the constraints involved in its fabrication and placement – droplets present a flawless surface, and can be easily printed at desired locations on a surface. Thereby, this approach produces a straightforward increase in the focusing capabilities of a system by a factor that depends to first order on the liquid refractive index. In addition, contrary to immersion objectives, liquid micro-lenses can be used in a variety of optical systems, the focusing enhancement can be controlled by simply changing the liquid, and the small size of the lens minimizes possible absorption effects and even temporal dispersion in the case of irradiation by ultrafast laser pulses. Here, we investigate the optimal conditions of DALP and demonstrate the feasibility of this approach by creating nanopatterns on a polymeric surface with a feature size about one fourth of the processing wavelength.

## Results

### Principle of droplet-assisted laser processing

The basis of our approach can be found in the lensing effect that a drop of liquid produces on a surface. This phenomenon – clearly observed when a liquid falls on a screen, with the underlying pixels appearing magnified - can be explained by the curvature and refractive index of the liquid (n > 1), causing each drop to act as a plano-convex lens. In particular, DALP uses micrometer-size droplets whose contact angle with the surface is about 90° (almost perfect hemispherical shape). By placing such a droplet at the focal plane of an optical system, the effective numerical aperture of the system increases and, consequently, enhancement in lateral as well as axial resolution is achieved^24^. Most importantly, droplet focusing is carried out without introducing spherical or coma aberrations. This is a consequence of the hemispherical nature of the droplet – light is focused at the plane lying just underneath the flat surface, at a position corresponding to the first aplanatic point of a sphere^25^.

To better understand focus enhancement induced by a micro-droplet, we simulated light focusing through an optical system with and without droplet. In particular, we considered a lens doublet as the focusing element (N.A. 0.27) and solved the diffraction propagation equation for a Gaussian beam (1027 nm wavelength). The colormap intensity plot at the vicinity of the system focus without micro-droplet is presented in Fig. 2A. The beam displays the characteristic features of a focused Gaussian beam. In particular, intensity at the focal plane presents a Gaussian profile along the radial direction, with a measured full-width at half maximum (FWHM) of 1.48 μm (Fig. 2C). Regarding beam intensity along the propagation direction (Fig. 2D), the beam presents the parabolic profile typical of Gaussian beams. The corresponding Rayleigh range, characterized by a decrease in intensity of a factor of 2, is equal to 6 μm. Notably, the focusing characteristics of the optical system drastically change when a hemispherical micro-droplet (n = 1.4, 20 μm in diameter) is placed at the focal plane, as shown in Fig. 2B. In this case, the lensing effect induced by the droplet produces stronger focusing, with an enhancement in intensity of about a factor of 2. In addition, the radial plot profile of Fig. 2C reveals a FWHM at focus of 1.05 μm, a factor of 1.4 smaller than without droplet. This value corresponds to the refractive index of the droplet, and it is in good agreement with theoretical work on SILs^26^. In fact, the droplet is expected to produce an increase in the effective numerical aperture of the system equal to the lens refractive index. Assuming no absorption in the droplet, the enhancement in intensity can be directly correlated to the decrease in spot size – the focusing area scales as the square of the radius, and hence the increase in intensity is 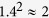. The micro-droplet also constrains the focus extent along the axial direction (Fig. 2D). In this case, the Rayleigh range is 3 μm. This is also consistent with the increase in numerical aperture of the system, since the Rayleigh range scales as the inverse square of this parameter^27^. Simulations performed with hemispherical droplets whose diameter ranges from 20 to 200 μm do not present significant differences, and in all cases the droplet produces an enhancement in the system numerical aperture proportional to the liquid refractive index.

### Implementation of DALP

**Figure 1:**
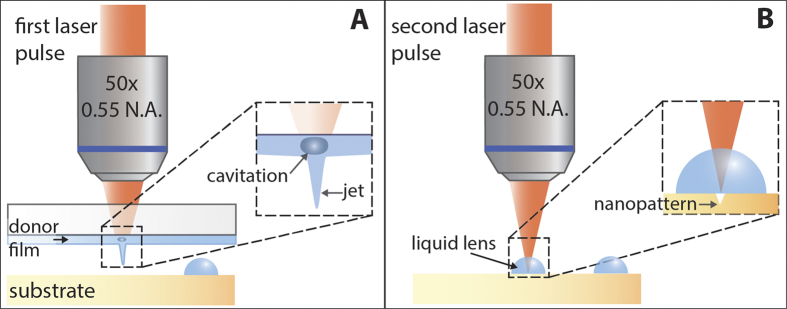
Schematic of the droplet-assisted laser processing approach. (**A**) A liquid droplet is printed on top of the surface to be patterned by using LIFT. (**B**) After removal of the donor film, the same laser source is focused through the droplet, which acts as a liquid lens. As a consequence, nanopatterns can be fabricated.

**Figure 2:**
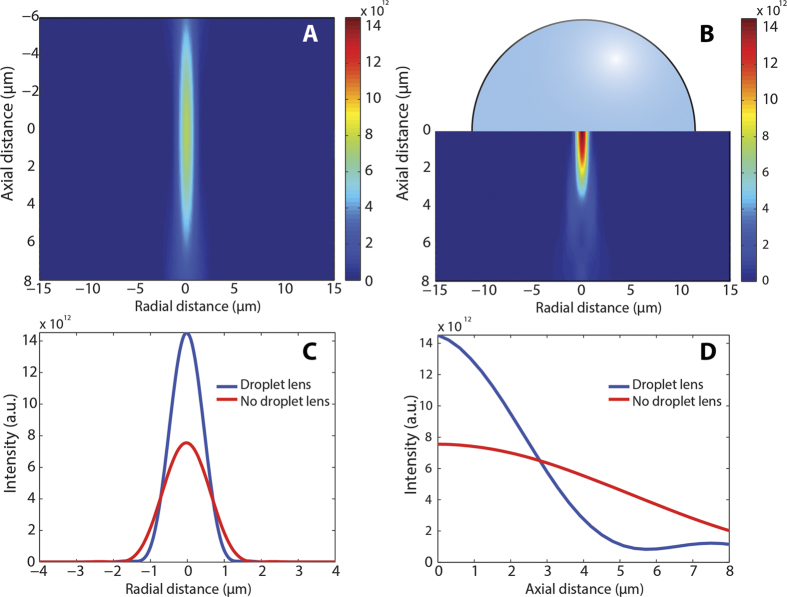
Simulation of the intensity and focusing enhancement produced by droplet-assisted laser processing. (**A**) Colormap plot of the intensity distribution of a Gaussian beam focused through a 0.27 N.A. objective, and (**B**) corresponding colormap plot after a hemispherical droplet (n = 1.4) has been placed at the laser focus. The liquid lens results in about a factor of 2 intensity enhancement. (**C**) Plot of the beam intensity versus radial distance at focus. In the case of using a liquid lens, the FWHM is decreased by a factor corresponding to the lens refractive index. (**D**) Plot of the beam intensity versus axial distance along the optical axis. The system depth of field is reduced when using the liquid lens.

The successful implementation of DALP requires a method capable of printing hemispherical droplets with controlled size at precise locations on a surface. Here, we used laser-induced forward transfer or LIFT to achieve this goal, but other methods such as inkjet printing^28^ could be valid as well. LIFT is a laser-direct writing technique based on transferring material from a donor film to a substrate placed in close proximity to it^23^ (Fig. 1A). In the case of using a liquid film, laser pulses induce the formation of a cavitation bubble in the liquid that evolves into a jet propelling a fraction of the material toward the substrate^29^,^30^. When the jet contacts the substrate, a droplet is formed^31^. Interestingly, droplets with diameters ranging from microns to sub-millimeters can be obtained by simply modifying the laser pulse energy, which renders this approach optimal for DALP. Contrary to inkjet printing^28^, the nozzle-free nature of LIFT makes this technique suitable for printing liquids with a wide range of rheological properties. In addition, the use of LIFT for DALP is further justified by the possibility of using the same laser source for both droplet lens printing as well as material processing.

A crucial aspect of DALP is the shape of the micro-droplet. Failing to achieve hemispherical droplets inevitably results in spherical aberrations and the consequent loss of optical performance. Therefore, it is necessary to assure an equilibrium contact angle between droplet and surface of 90°. Provided a hydrophobic surface, contact angle can be adjusted by adding a surfactant to the liquid. This is the approach we used in the current experiment, where the contact angle between polydimethylsiloxane (PDMS) and a mixture of water and glycerol (n = 1.5), typically around 110°, was decreased to 90° by adding a surfactant (Supplementary Figure 1). In the case of patterning an initial hydrophilic surface, a hydrophobic coating must be first applied. By using spin-coating or other chemical deposition processes, thin films below 10 nm can be obtained^32^, with minimal impact on the optical performance of DALP. Besides the need for contact angle control, there is a second factor that plays a crucial role in the formation of a hemispherical droplet: the balance between gravitational and surface tension forces – gravity tends to pull the center of the droplet disrupting its spherical shape^33^. The ratio between these forces can be expressed using a dimensionless number, the Bond number:


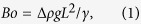


where 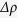 is the density difference between drop and air, g is the gravity constant, *γ* is surface tension and L is a characteristic length of the droplet (diameter, in this particular case). For *Bo* ≤ 0.01 gravitational effects can be neglected^33^, and the droplet retains a perfect spherical shape. When using aqueous solutions, such a value is reached for diameters below 300 μm, which justifies the need for printing micrometer-size droplets in order to obtain aberration-free liquid lenses.

Once micro-droplets with a hemispherical shape are deposited on a surface, sub-wavelength nanopatterning is possible by focusing laser pulses through the droplets. At this point, there are two different modes to implement DALP. The first one consists of firing a single laser pulse for each droplet. Any potential deformation or even volatilization that the droplet may suffer as a consequence of laser irradiation becomes irrelevant – each droplet/lens is used only once. In fact, for sub-picosecond pulses, volatilization would occur well after the laser pulse is over^34^. This approach works best when small features with large separations are required, since the minimum distance between spots is initially determined by the droplet diameter. By repeating the printing process, the separation between features can be reduced, but this process becomes cumbersome. Alternatively, it is possible to fabricate structures arbitrarily separated by using a single droplet and scanning the laser beam across it. This provides a direct approach for fabricating user-defined structures, including continuous nanopatterns, but questions remain about the performance of the lens off center, as well as the potential droplet deformation caused by multiple firing.

### Experimental characterization of DALP

**Figure 3:**
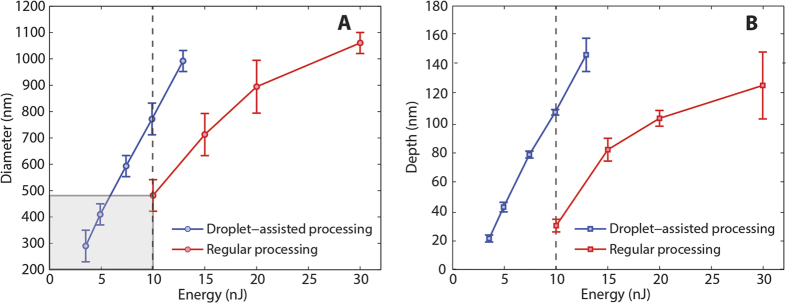
Plot of hole diameter versus energy for DALP and regular processing. The deep sub-wavelength patterning area accessible only by DALP is shown in gray. (**B**) Plot of hole depth versus energy. Notably, for an energy of 10 nJ, holes obtained by DALP are about 3 times deeper.

**Figure 4:**
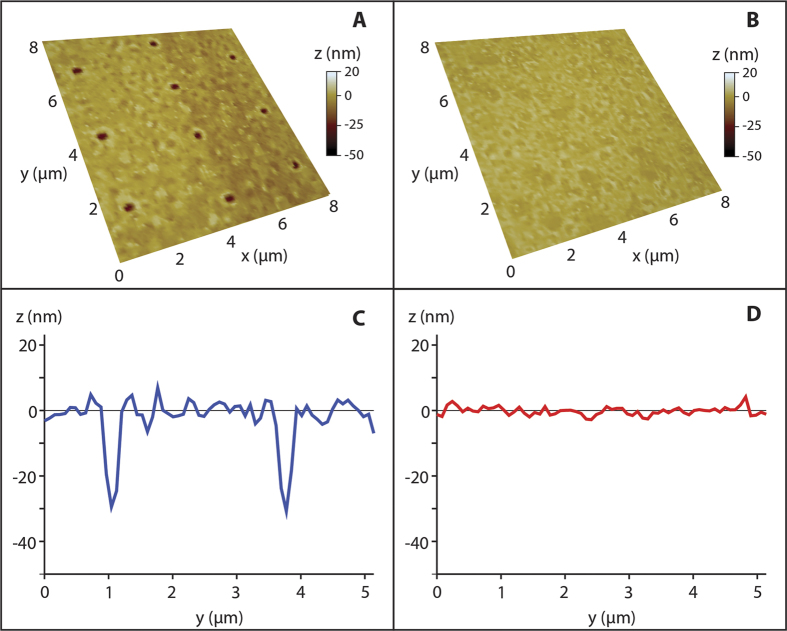
Characterization of droplet-assisted laser processing versus regular processing. (**A**) AFM image of 280 nm diameter holes obtained by single-shot ablation at an energy of 3.5 nJ using DALP with a droplet size of 100 μm. (**B**) Morphology of the same surface irradiated using 3.5 nJ using regular processing. No modification is observed (**C**) Line scan corresponding to Fig. 3A, and (**D**) to Fig. 3B.

To demonstrate the feasibility of DALP, we first laser printed an array of droplets on PDMS using an energy of 8.5 μJ (1027 nm wavelength, 450 fs pulse duration). The corresponding laser fluence was 2.7 J/cm^2^ (laser was defocused). Well-defined droplets with an average diameter of 100 μm were obtained, with a standard deviation of only 4 μm (Supplementary Figure 2). Such droplet control assures good reproducibility to our approach. Next, we removed the donor film used in LIFT and moved axially the planar surface of the droplet to match the objective focal plane. We then fired 10 shots at the vicinity of the droplet center, each spaced 2 μm from one another. We repeated this experiment for different laser pulse energies, using a different droplet for each energy (range of 2 to 30 nJ, which corresponds to fluences from 80 to 1170 mJ/cm^2^). Plots of the spot diameter and depth measured by atomic force microscopy (AFM) are presented in Fig. 3 as a function of laser pulse energy. For comparison, the results obtained with regular laser processing (no droplet) are also included. Notably, given a laser pulse energy, the spot diameter is larger and the depth is greater with DALP. This can be explained by the stronger focusing produced by the droplet and the consequent increase in the effective laser fluence. In fact, by using a moderate numerical aperture objective (N.A. 0.55 in this experiment), energies ranging from 3.5 to 10 nJ can be used to ablate PDMS. The corresponding laser fluences are 140 and 390 mJ/cm^2^, below the ablation threshold for PDMS without a droplet (390 mJ/cm^2^ in the current experiment, in agreement with values found in literature^35^). Such low energies open the door to use DALP with low power lasers or directly from laser oscillators. More importantly, at these energies (sub-threshold fluence) deep sub-wavelength patterning is possible. In this way, spot sizes as small as 280 nm were obtained for 3.5 nJ pulses, about one fourth of the processing wavelength (Fig. 4). This is almost a factor of 1.8 smaller than the minimum spot size obtained in this experiment with regular laser processing (490 nm). Such a reduction in feature size above the liquid lens refractive index (1.5), is caused by the threshold effect characteristic of laser ablation and the nonlinear relationship between beam waist and laser pulse energy^36^. Therefore, even if the laser beam waist is reduced by a factor equal to the lens refractive index, nonlinearities in light-matter interaction enables one to use DALP to further reduce spot size.

**Figure 5:**
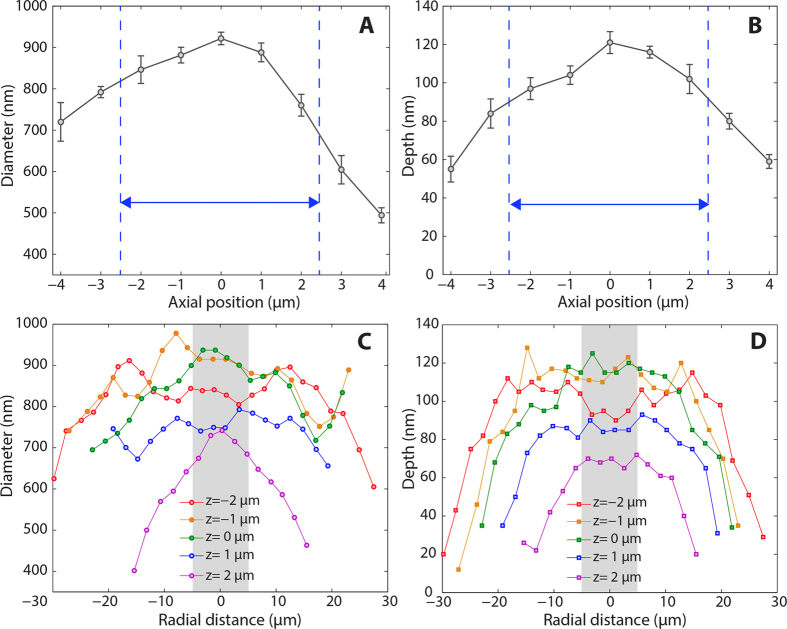
Alignment tolerance of droplet assisted processing. Plot of the diameter (**A**) and depth (**B**) of holes ablated in the center of the droplet at different axial positions. The presence of the droplet provides enhanced focusing but does not modify the axial working range suitable for uniform processing. Plot of the diameter (**C**) and depth (**D**) of holes ablated using DALP at different radial positions within a droplet. For a distance corresponding to about 10% of the droplet diameter, the hole size and depth remains constant indicating that aberration-free nanopatterning is possible.

Figure 5A,B show the change in spot diameter and depth as the beam is focused through the droplet at different axial positions. Such characterization is of pivotal importance for the technical implementation of DALP. Indeed, accurate z-focus control is a classic problem in laser patterning: real-world surfaces are non-flat and can present warpage or texture, which typically require costly and time consuming methods capable of adjusting the focal plane relative to the surface for uniform processing to occur^37^. This problem is aggravated when high spatial resolution is desired, i.e. in high numerical aperture systems or near-field super-resolution techniques, where the short depth of field (DOF) inherent of these approaches constrain their use to the de facto processing of flat surfaces. In contrast, DALP tolerates an axial shift of the focal plane as high as 5 μm (Fig. 5A,B). Within this range, ablated holes at an energy of 12 nJ present a diameter and depth variation of only 10% with respect to the mean value. Such axial processing range is comparable to the DOF of the optical system without droplet (Supplementary Figure 3). Therefore, the enhancement in lateral resolution produced by DALP does not impose stronger requirements on axial focus control. In other words, DALP increases the effective numerical aperture of a focusing system without altering its original working distance or DOF. This can be understood by the theory of hemispherical SILs: as long as the planar surface of the lens is within the DOF of the focusing objective, strong focusing and deep sub-wavelength processing can be obtained without aberrations. In addition, the critical distance between lens and surface is maintained constant in DALP – the droplet is always in contact with the surface to be patterned, effectively making DALP a self-positioning approach.

The focusing performance of a liquid micro-lens off center is presented in Fig. 5C,D. An array of holes was prepared by laterally scanning the laser beam across a 100 μm droplet. The plots of the ablated hole diameter and depth versus radial position (Fig. 5C,D) show variations for these parameters of less than 5% (maximum – minimum values) up to a distance of about 5 μm off the lens center for all axial positions analyzed. This radial distance corresponds to about 10% of the initial droplet diameter. Such a value is in agreement with numerical simulations for different droplet sizes (Supplementary Figure 4), where in all cases a change of less than 2% in the FWHM of the focused beam is obtained for up to 10% of the droplet diameter – in fact, due to laser energy fluctuations the experimental values are expected to present a slightly higher variability than theory. As the beam is focused off the lens axis, the diameter values starts to vary significantly while the hole depth tends to decrease, which is consistent with the presence of aberrations at these positions. Consequently, it is possible to use DALP to write nanopatterns within a single droplet, but the irradiated area must remain relatively close to the lens center. Considering the limit in droplet diameter imposed by gravity (Bond number) for most common fluids, ∼100 μm droplets present a suitable trade-off between preservation of hemispherical shape – absence of aberrations – and area that can be patterned (10 × 10 μm^2^).

Potential distortions within a droplet caused by laser firing are also an important aspect to consider. The nature of such distortions can be diverse, largely depending on laser pulse energy or laser repetition rate. In this particular experiment, we observed the formation of micrometer-size cavitation bubbles for energies exceeding 15 nJ (fluence of 59 mJ/cm^2^). Such phenomenon can be caused by the use of energies above the optical breakdown in the liquid^38^, or by by-products of the ablative process^39^. Most bubbles seem to disappear after around 1 second, but the scattering induced by a bubble already imposes a limitation in the repetition rate of DALP: about 1 Hz for E > 15 nJ for this current experiment. For energies below 15 nJ and repetition rates below 5 Hz we observed no distortion in the droplet shape nor any cavitation effects. However, higher repetition rates resulted in non-circular ablated holes. We attribute this effect to the heating induced by the ablation of the polymer, and the consequent change in refractive index in the liquid that caused aberrations during droplet focusing. In particular, when firing two consecutives laser pulses (repetition rate higher than 5 Hz) at slightly different positions, the thermal effects induced by the first shot can laterally perturb the second one, breaking the circular symmetry of the focal spot and causing coma aberration. Using higher energies or higher repetition rates resulted in a significant aggravation of these effects, eventually producing a change in the droplet shape or volume, or even its total volatilization. In such instances, though, it is still possible to use DALP using a single pulse for each droplet.

### Nanopatterning

**Figure 6:**
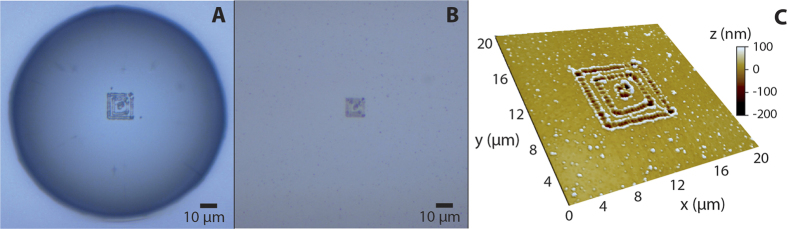
Nanopatterning of concentric squares. Optical micrograph of a pattern fabricated with a 100 μm droplet, (**A**) with the droplet still in place and (**B**), without droplet. The drop produces a magnification of about a factor of 1.5. (**C**) AFM characterization of the pattern.

The nanopatterning capabilities of DALP are presented in Fig. 6. In this case, we prepared 3 concentric squares using a 100 μm droplet and 5 nJ pulses. Notably, the formation of continuous features with a width of 200 nm is feasible by pulse overlapping (shot-to-shot distance of 100 nm in the current experiment). After laser irradiation and with the droplet still in place, the pattern can be clearly distinguished upon examination in an optical microscope (Fig. 6A). Washing out the droplet does not modify the pattern, which is still visible by optical microscopy (Fig. 6B). However, a simple comparison of the 2 optical micrographs reveals the lensing effects of the droplet. Indeed, the pattern imaged through the droplet appears magnified by a factor of about 1.5. This value is close to the refractive index of the liquid. In addition, the resolution is also enhanced by the droplet – nearby features are only distinguishable by droplet imaging. A statistical analysis of the pattern from AFM characterization (Fig. 6C) shows an average line width of 350 nm and depth of 50 nm, with a standard deviation of 30 nm and 5 nm, respectively. It is important to note that the lack of parallelism in the fabricated structure is not a fundamental limitation of DALP but is caused by the limited position accuracy of the mechanical stages.

## Discussion

In this paper, we have successfully demonstrated that liquid micro-droplets can be used to enhance the focusing capabilities of a laser direct-writing system, and produce features with a size down to 280 nm at sub-threshold fluences, about one fourth of the processing wavelength. We have implemented our approach by first laser-printing hemispherical droplets at user-defined positions on a surface, and subsequent firing through the droplet. Each droplet acts as a lens, whose optical performance depends on the refractive index of the liquid in a way analogous to a solid immersion lens. The axial position of the droplet with respect to the focal plane of the optical system does not significantly compromise the outcome of our approach as long as the plane surface of the droplet is within the depth of field of the focusing objective. Furthermore, continuous nanopatterns can be written within a single droplet by scanning the laser in the XY direction at low laser fluences and low repetition rates. No artifacts or aberrations are induced off the droplet center up to about 10% of the droplet diameter. In addition, droplet-assisted laser processing could further improve resolution by using high refractive index liquids, including polymers or nanoparticle-doped solutions^40^. DALP is easy to implement using any laser-direct writing system, it can be performed in ambient conditions and it obviates the need for precision axial z-focus control or other constraints typical of current nanopatterning approaches. By combining optics and fluidics, DALP provides researchers with a powerful tool capable of overcoming the traditional diffraction barrier, extending the well-known advantages of lasers to deep sub-wavelength patterning.

## Methods

### Laser direct-writing system

A Yb:KYW femtosecond laser (Amplitude Systemes S-pulse) with a wavelength of 1027 nm and a pulse duration of 450 fs was used in single shot mode. The beam profile was Gaussian with M^2^ = 1.35. The laser pulse energy was controlled by means of a variable optical density filter, and the energy of each pulse was measured by an energy meter (Ophir PD10-SH). The laser irradiation was focused onto the sample using a 50x, 0.55 NA long-working distance objective (Mitutotyo M Plan Apo 50X). The sample was positioned on a XYZ mechanical translation stage with a minimum step size of 0.5 μm (Physik Instrumente TM M-414) aligned perpendicular to the focusing objective. Real time visualization of the ongoing process was carried out by a CMOS camera (Thorlabs DCC1645C) thanks to a dichroic mirror that reflected IR (1027 nm laser) and transmitted the visible spectrum.

### Sample preparation

A solution of polydimethilsiloxane (PDMS) dissolved in ethyl acetate (5% v/v) was drop-casted on top of a silicon wafer. After solvent evaporation, a PDMS film with a thickness of 200 nm was obtained. Thickness control could be achieved by changing solvent concentration, but 200 nm presented the highest uniformity. It is important to note that the thickness of the polymeric layer cannot be smaller than a certain value (which depends on the optical absorption of the polymer) or otherwise ablation of the underneath surface occurs. In this case, the PDMS layer acts as a coating that provides the appropriate contact angle between droplet and surface to be patterned.

### Laser printing

The donor film used in LIFT was prepared by blade coating a solution of water and glycerol (50% v/v) with 1% sodium dodecyl sulfate on top of a microscope slide covered with a 50 nm thick titanium film. The titanium film was used as the absorbing layer. No presence of titanium on the printed droplets was observed. However, in order to completely rule out potential contamination by the absorbing layer, it would be possible to use a volatile organic absorbing layer^41^ or even directly print from a liquid reservoir by using high intense pulses without any absorbing layer^42^. The thickness of the liquid film obtained was about 10 μm estimated from a weight measurement. The donor film was placed on top of the PDMS surface – liquid facing PDMS – at a distance of 100 μm using spacers. The donor-PDMS substrate system was then moved to the XYZ stage to initiate the printing process. Due to the low vapor pressure of the water and glycerol solution, the donor film remained stable over a period of about 1 hour. For the same reason, the diameter of the printed droplets changed very slowly over time while its hemispherical shape was in all cases maintained (contact angle of about 90°), providing an effective processing window time for DALP of about 30 minutes.

### Optics simulation

The physical optics propagation (POP) package from Zemax (Radiant Inc.) was used to solve the diffraction equations for an optical system consisting of a lens doublet with an estimated numerical aperture of 0.27. A Gaussian beam with a wavelength of 1027 nm was launched through the system, and the intensity distributions at different axial positions were measured. To simulate the effects of DALP, the simulations were repeated with a hemispherical lens with refractive index of 1.4 placed at the system focus.

### Sample characterization

Optical micrographs of the fabricated patterns were captured with a commercial microscope (Carl-Zeiss Axio Imager). AFM characterization was carried out in non-contact mode using a pyramidal tip with size of 14–16 μm (AppNano AFM probes ACTA), in an AFM system (Park Systems XE-70). Contact angle measurements were performed using a goniometer system (KSV CAM 200 Optical Tensiometer). In all cases, MATLAB was used for data analysis.

## Additional Information

**How to cite this article**: Duocastella, M.D. *et al.* Sub-wavelength Laser Nanopatterning using Droplet Lenses. *Sci. Rep.* **5**, 16199; doi: 10.1038/srep16199 (2015).

## Supplementary Material

Supplementary Information
