# Supplementary material for: Sub-wavelength Laser Nanopatterning using Droplet Lenses
Source: Sci Rep. 2015 Nov 6;5:16199. doi: 10.1038/srep16199 (PMC4635425; doi:10.1038/srep16199)
Supplement: Supplementary Information [file srep16199-s1.doc]

**Sub-wavelength Laser Nanopatterning using Droplet Lenses**

Martí Duocastella^1*^, Camilo Florian^2^, Pere Serra^2^ and Alberto Diaspro^1^

^1^Nanoscopy, Istituto Italiano di Tecnologia, Via Morego 30, 16163 Genova, Italy

^2^Departament de Física Aplicada i Òptica, Universitat de Barcelona, C/Martí i Franqués 1, 08028 Barcelona, Spain

*E-mail: marti.duocastella@iit.it

**Supplementary Figures**


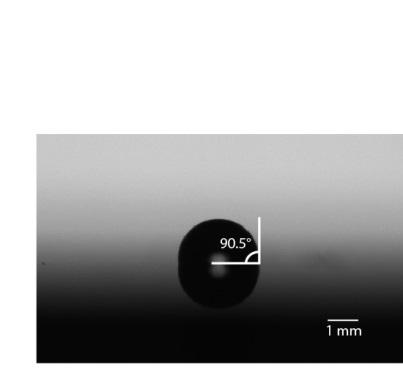


Supplementary Figure 1. Contact angle of a droplet on top of the surface to be patterned, with a contact angle of about 90°.


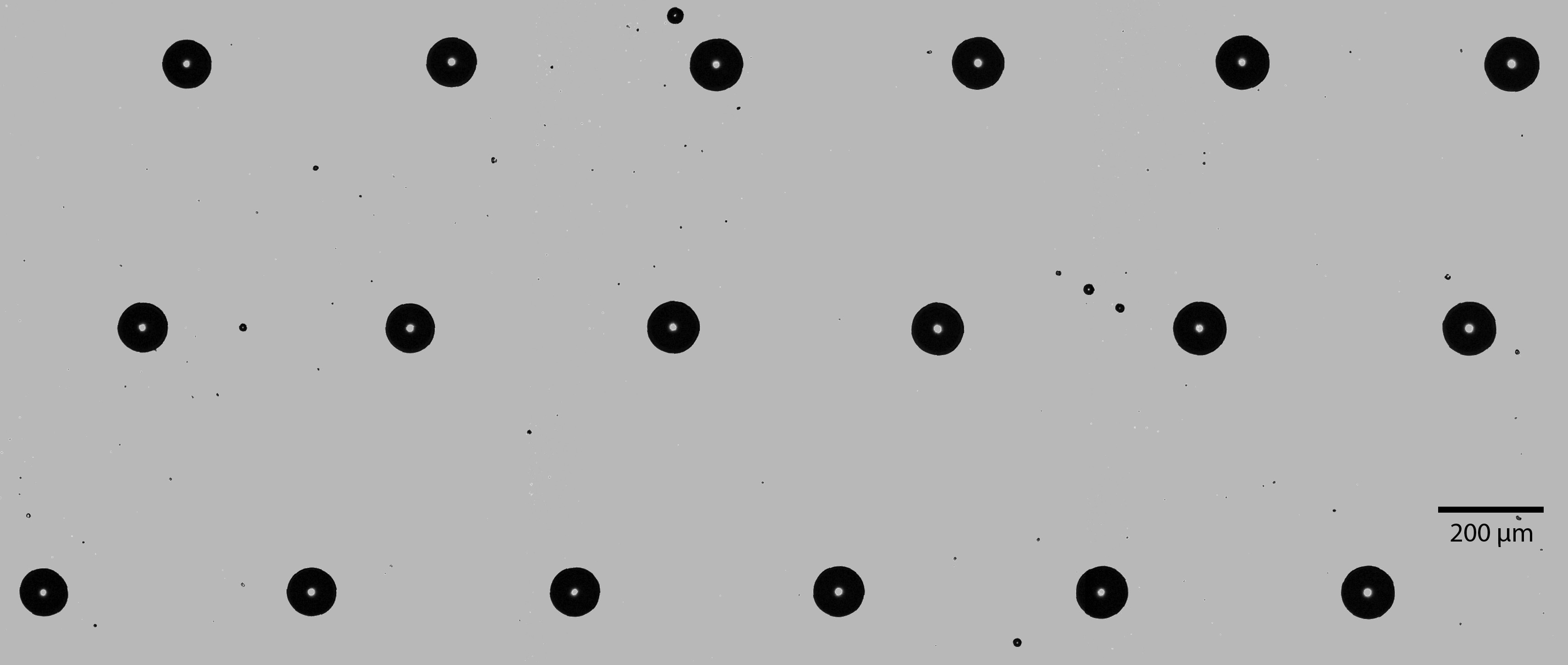


Supplementary Figure 2. Array of 100 µm droplets printed using laser-induced forward transfer (LIFT) at an energy of 8.5 µJ. The standard deviation of the droplets is 4 µm.


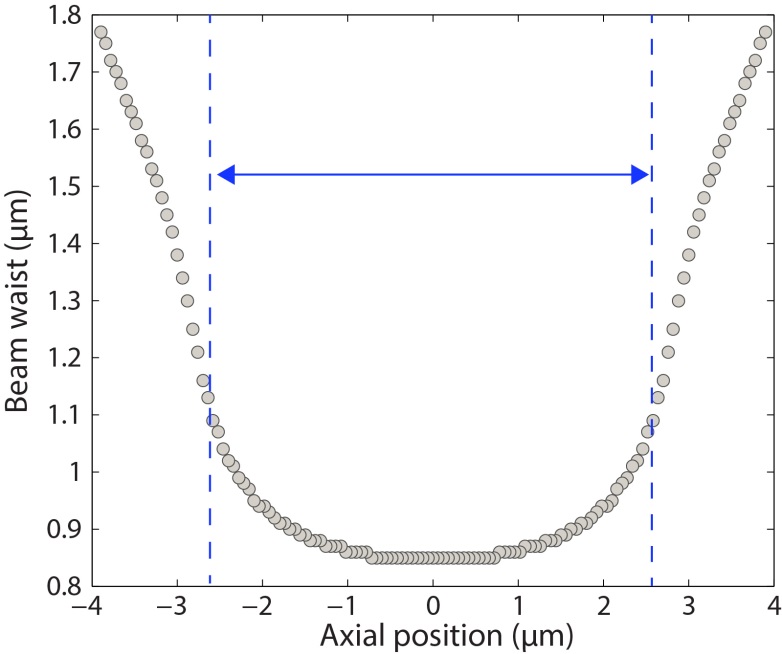


Supplementary Figure 3. Plot of the experimentally measured laser beam waist versus axial position for the optical system without micro-droplet. The depth of field (DOF), defined as twice the Rayleigh range - where the minimum beam waist increases a factor of $\sqrt{2}$- is about 5 µm, in good agreement with the axial tolerance found in DALP.


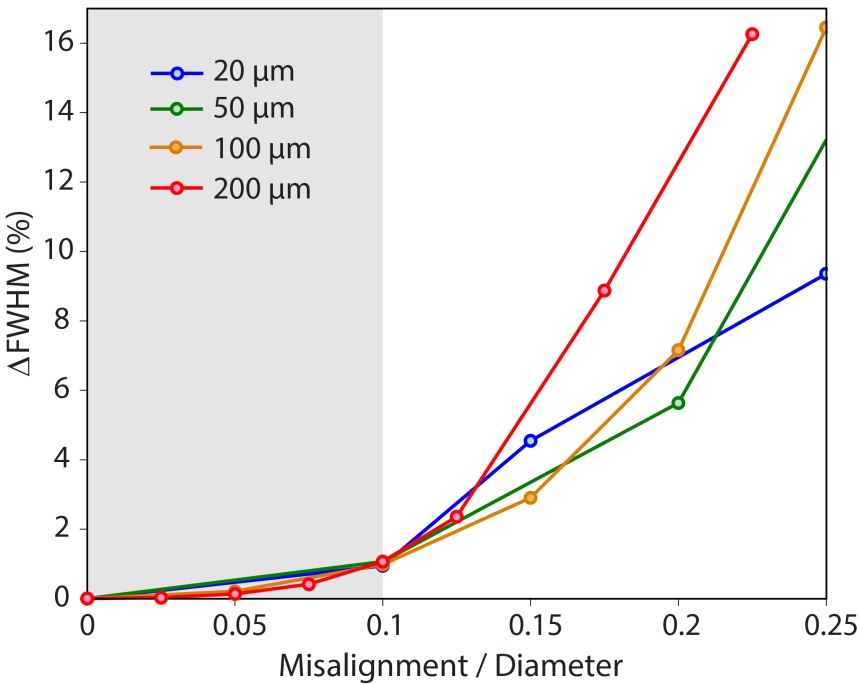


Supplementary Figure 4. Simulations of radial misalignment tolerance in DALP. Plot of the changes in FWHM of the intensity distribution at the focal plane of a beam focused through an objective and a droplet (n=1.4) versus the shifting of the beam optical axis relative to the lens center normalized by lens diameter. Notably, the beam can be shifted up to 10% with respect to the lens center without significant width changes.
